# Fracture Resistance of Zirconia Surveyed Crowns With Digitally Designed and Hand-Modified Occlusal Rest Seats

**DOI:** 10.7759/cureus.64423

**Published:** 2024-07-12

**Authors:** Nabeel M Munshi, Mohammed Alsufayri, Adham Alzahrani, Carlos A Jurado, Maher S Hajjaj, Mosa Altassan, Saeed J Alzahrani

**Affiliations:** 1 Department of Oral and Maxillofacial Prosthodontics, Faculty of Dentistry, King Abdulaziz University, Jeddah, SAU; 2 Department of General Dentistry, King Abdulaziz University, Jeddah, SAU; 3 Department of General Dentistry, College of Dentistry, University of Tennessee Health Science Center, Memphis, USA; 4 Department of Restorative Dentistry, King Abdulaziz University, Jeddah, SAU

**Keywords:** mechanical testing, zirconia cad-cam crown, monolithic zirconia, removable partial denture, milling, fracture resistance, zirconia crowns, surveyed crowns

## Abstract

Background

In light of the trend of using zirconia crowns, clinicians will likely face abutment included in removable partial dentures (RPD) designs with existing zirconia. However, the decision to replace the existing crown with a surveyed crown or modify the existing crown to accept the RPD is unclear. To the best of our knowledge, there is a lack of literature on the effect of preparing a rest seat on the existing monolithic zirconia crown in the patient’s mouth on the fracture resistance of the crown. Therefore, in this study, we aimed to evaluate the fracture resistance of computer-aided design/computer-aided manufacturing (CAD/CAM) zirconia surveyed crowns with digitally designed rest seats and hand-modified rest seats.

Methods

Thirty CAD/CAM zirconia surveyed crowns were digitally designed and fabricated and divided into groups (n=10 per group) as follows: Group 1 comprised surveyed crowns with no occlusal rest seat; Group 2 comprised surveyed crowns with a digitally designed mesial rest seat; and Group 3 comprised surveyed crowns with a hand-modified mesial rest seat. Then, with all the crowns cemented to metal dies, the specimens were subjected to a fracture resistance test using a universal testing machine (Model 8501 Instron, Norwood, MA, USA).

Results

Surveyed crowns without any rest seat and those with digitally created and hand-modified rest seats displayed different fracture resistances: crowns with no rest seat offered the highest fracture resistance (5831 ± 895.15 N), followed by those with a digitally designed and milled rest seat (5280 ± 1673.33 N). Crowns with a hand-modified rest seat provided the lowest fracture resistance (4976 ± 322.5 N). Based on our results, surveyed crowns without a rest seat displayed higher fracture resistance than those with a rest seat.

Conclusion

The fracture resistance of crowns with a digitally designed and milled rest seat was statistically similar to that of control crowns with no rest seat, whereas hand-modified rest seats significantly reduced the fracture resistance of surveyed zirconia crowns.

## Introduction

The prevalence of complete edentulism has decreased in the past few years due to the increased use of fluorides, better access to oral healthcare, and advancements in treatment modalities [1]. However, partial edentulism remains prevalent, with as many as one in five patients complaining of partial edentulism in some areas [2]. Fortunately, there is more than one alternative treatment to replace missing teeth depending on oral condition and patient demand, such as dental implants and fixed or removable dental prostheses (FDP and RDP, respectively). Removable partial dentures (RPD) is arguably the best available treatment option in terms of systematic disease that prevents implant treatment options, the use of remaining supporting abutments, treatment time, and financial burden [3]. Several requirements must be met for successful implementation of the RPD, including the presence of good abutment teeth to provide support from an intact occlusal surface and adequate retention from the buccal or lingual surfaces. If the abutment tooth cannot meet these requirements, it must be restored with a surveyed crown [3].

As an abutment for a RPD, a fixed restoration on a tooth is considered one of the most complicated restorative procedures in prosthodontics [4]. In RPD abutments, the single crown, which has been referred to as a surveyed crown, is used to enhance the path of insertion and occlusal relationship, as well as support and retain the RPD framework [4]. Thus, the single crown helps to adjust occlusal plane discrepancies, create a proper rest seat area, and create adequate retentive undercuts for direct retainers and ideal guide-plane surfaces [4]. The occlusal rest seat of the RPD provides longitudinal support, allows occlusal loads to be transmitted across the abutment tooth’s long axis, and prevents the denture base from impinging on the supporting soft tissues [3]. There are a variety of rest seat designs available to clinicians, depending on the design of the RPD. The cingulum rest is most commonly used for the anterior teeth, whereas the occlusal proximal rest is the most common design for the posterior teeth [5].

Many different materials used to create surveyed crowns are found in the literature [5]. All-metal crowns using gold and gold alloys were the first materials to be used as surveyed crowns. Gold-based crowns have several advantages, such as conservative preparation, biocompatibility, adequate strength, and great marginal fit, but they also suffer from poor esthetics and high cost [6]. Porcelain fused to metal (PFM) crowns are now the standard for fabricating surveyed crowns owing to their good mechanical properties, marginal adaptation, and acceptable biological quality needed for periodontal health. However, the drawbacks of using PFM crowns, including porcelain chipping, extensive tooth preparation, esthetics, and the increased cost of noble metals, have recently necessitated the search for alternative materials [6]. All-ceramic survey crowns have been suggested due to their high strength, ease of fabrication with computer-aided design/computer-aided manufacturing (CAD/CAM) (compared to casting and layering of PFM crowns), and esthetic properties [7]. Zirconia has excellent mechanical and esthetic properties and has shown relatively good short- and medium-term survival rates [8].

Zirconia material for the fabrication of indirect restorations is available in different types or generations depending on the percentage of yttria present [9-12]. Yttria is added to the zirconia powder to stabilize the tetragonal phase, which is the most desirable phase mechanically [9]. Hence, the full scientific name of the material is yttria-stabilized tetragonal zirconia polycrystal (Y-TZP), which includes the stabilizer agent and the phase at which zirconia stabilizes [10]. The type of zirconia refers to the mol% of yttria added to its composition [10]. There are five types of zirconia, three of which are made from a pure single layer, i.e., 3Y-, 4Y-, and 5Y-zirconia [9]. The strength of the zirconia decreases significantly as the compositional percentage of the yttria increases; the average strength of 3Y-zirconia is 900-1200 MPa, whereas the maximum strength of 5Y-zirconia is 600 MPa [9]. However, increasing the percentage of yttria in the composition improves the translucency of the zirconia [11]. Contrast ratio, as a parameter to measure the transparency of materials, decreases (i.e., improves) from 0.90 to 0.72 among 3Y-zirconia and 5Y-zirconia [11]. The remaining two zirconia types have been recently introduced as multi-layered Y-TZP 5Y with 3Y or 5Y with 4Y zirconia. These multi-layered zirconia types are advantageous in that they possess the strength of 3Y- or 4Y-zirconia and the esthetic of 5Y-zirconia [9]. 

Due to the widespread use of zirconia restorations, several clinical reports have demonstrated the successful use of zirconia crowns as surveyed crowns [12]. In national dental practice surveys in the United States, 30% of dentists prefer zirconia as the first material selected for posterior teeth [13]. In a clinical study, the outcome of veneered zirconia single crowns supporting and retaining RPDs after four years was investigated. The most common complication was fracture of the veneering porcelain, whereas the rarest was fracture of the occlusal rest [14]. The use of monolithic zirconia, instead of veneering porcelain, can address this clinical complication.

Several in vitro studies investigated the load to fracture of surveyed zirconia crowns with different specifications and parameters. One of these studies investigated the fracture resistance of computer-aided design/computer-aided manufacturing (CAD/CAM) monolithic zirconia surveyed crowns using mandibular canines with two different cingulum rest seat designs and found that increasing the radius of curvature led to a statistical increase in fracture resistance [15]. Another investigation compared the load to fracture of surveyed and conventional monolithic zirconia crowns with two different occlusal thicknesses (0.5 and 0.75 mm). The results showed that the thicker occlusal rest had a higher fracture resistance than the thinner one. However, all groups of zirconia crowns fracture at a higher value than the maximum occlusal load values [15]. Chaturvedi et al. [16] investigated the fracture resistance of CAD/CAM all-ceramic surveyed crowns with different occlusal rest seat designs on monolithic CAD/CAM surveyed crowns. The load-to-fracture test showed that surveyed crowns with a narrow-base occlusal rest seat design (i.e., one-third of the buccolingual width) had statistically significantly higher fracture resistance than those with a wide occlusal rest seat design (i.e., two-thirds of the buccolingual width).

According to the literature, monolithic zirconia-surveyed crowns with milled occlusal rest seats can be used to support RPDs. However, clinicians encounter many clinical scenarios in which abutments can be used to support the RPD with an existing zirconia crown. Abutment preparation for the survey crown includes rest seat preparation to provide sufficient thickness for the crown materials and space for the occlusal rest to be seated without occlusal interference. In a laboratory study, a nonanatomic core with a 0.7-mm diameter was sufficient to withstand cycle fatigue stresses [17]. In addition, a recent study found that the fracture resistance of 1-mm-thick zirconia was higher than that of 1.5-mm-thick porcelain fused to metal [18]. Therefore, it is worth attempting to prepare the rest seat in the existing zirconia crown to save the patient financial and time costs.

Unfortunately, the decision to replace an existing crown with a surveyed crown or modify the existing crown to accept the RPD is often unclear. To the best of our knowledge, no literature has reported the effect of preparing a rest seat on the existing monolithic zirconia crown in the patient’s mouth or the influence of adding this preparation on fracture resistance. Therefore, the purpose of this in vitro study was to evaluate the effect of a manually prepared rest seat on the fracture resistance of a milled monolithic zirconia crown. Our null hypothesis was that there is no significant difference in load to fracture between the manually prepared occlusal rest seat on the zirconia crown and the surveyed zirconia crown with a milled occlusal rest seat.

## Materials and methods

**Figure 1:**
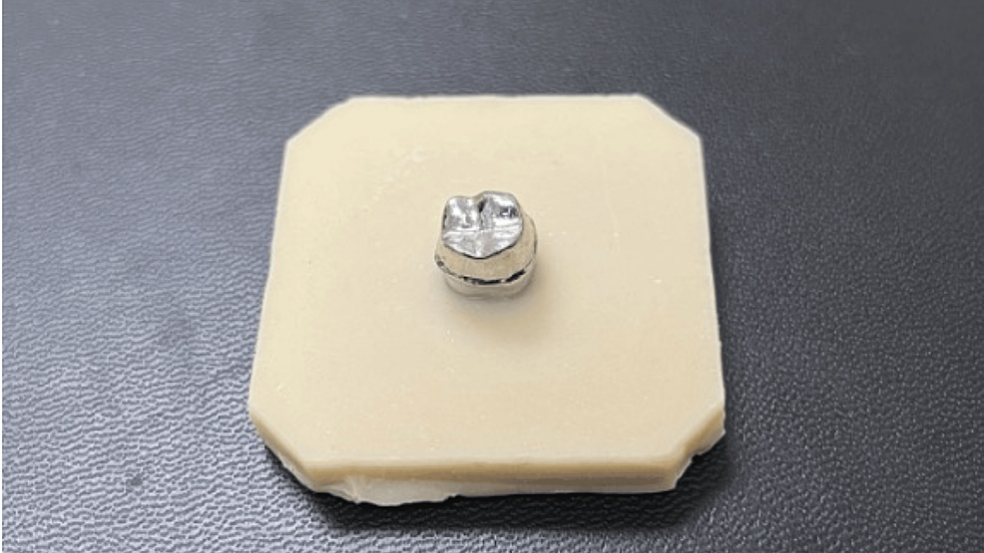
Metal die embedded in acrylic resin.

The study was conducted at the Faculty of Dentistry, King Abdulaziz University, Jeddah, Saudi Arabia. A mandibular first-molar plastic typodont tooth (Kavo Kerr, Biberach, Germany) was prepared to receive a full-coverage monolithic zirconia crown with a 1-mm circumferential chamfer finish line and a 1.5-mm occlusal clearance. A silicon mold was made for the prepared plastic typodont tooth. Then, molten wax was poured into the mold to create a wax pattern for the prepared tooth. After that, the wax pattern was invested, burned out, and casting done using nickel-based dental metal-ceramic casting alloy type 4 (4all, Ivoclar Vivadent, Schaan, Liechtenstein). The casting dies were retrieved from the investment, finished, and polished, and then placed in a putty mold to fabricate an acrylic base for handling purposes (Figure 1).

Three groups were included in the study design (n=10 per group): Group 1 received a fully contoured zirconia crown designed without any modification (control group), Group 2 received a zirconia crown with a mesial rest seat added to the design, and Group 3 received a zirconia crown with a hand-modified prepared rest seat. The rest seats in Groups 2 and 3 had the following specifications: half the distance from the mesial marginal ridge to the center of the tooth, one-third the buccolingual, and 1 mm in depth (Table 1).

**Table 1:** Group specifications.

Group	Specifications of the zirconia crown
Control	Designed fully contoured and after milling no modification added
Milled rest seat	Designed with a mesial rest seat and milled with rest seat
Hand-modified rest seat	Designed fully contoured and after milling hand-modified rest seat prepared on the crown

To standardize the manual rest seat preparation, the preparation was done by using a preparation guide jig made from self-curing acrylic resin (Pattern Resin, GC Cooperation, Tokyo, Japan). The burs used for preparation were high-speed, round, medium-grit diamond burs of size 1 mm (BRASSELER USA, Savanah, GA, USA). Then, preparation was finalized using high-speed, fine-grit, round, diamond burs (BRASSELER USA, Savanah, GA, USA). Copious water irrigation was used during preparation to avoid crack formation, and a periodontal probe was used for measurement. 

The metal dies were scanned with a laboratory scanner (KaVo ARCTICA AutoScan, Kavo Kerr, Biberach, Germany). All crowns were designed using CAD software (exocad GmbH, Darmstadt, Germany) and milled with monolithic zirconia 5Y-PSZ blocks (Cercon ht, Dentsply Sirona, Charlotte, NC, USA) using a laboratory milling machine (InLab MC X5, Dentsply Sirona, Charlotte, NC, USA). Then, the crowns were sintered in a ceramic furnace (Programat S, Ivoclar Vivadent, Schaan, Liechtenstein). 

All specimens were checked with a digital caliper to evaluate the remaining zirconia thickness, with a minimum of 1 mm in the rest seat area. Complete seating of the crowns was confirmed with no binding. All crowns were cemented on the metal dies using dual-cure self-adhesive resin cement (Breeze, Pentron Clinical Technologies, Wallingford, CT, USA). A thin layer of luting cement was applied to the fitting surface of the crowns, which were then seated on the metal dies with finger pressure, and any excess cement was cleaned. 

**Figure 2:**
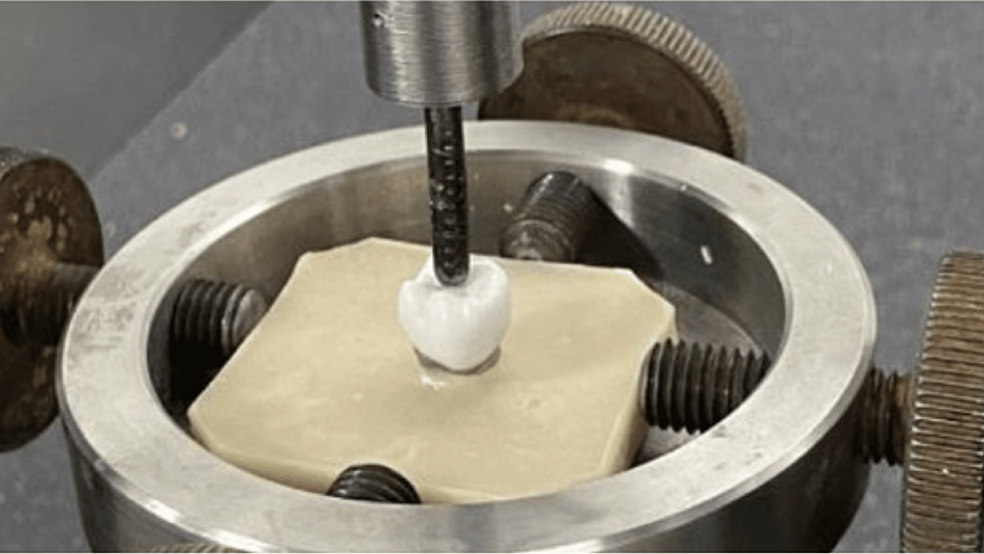
Crown subjected to a load to fracture using a universal testing machine.

After 24 hours of water storage at room temperature, the specimens were subjected to a fracture resistance test using a universal testing machine (Model 8501 Instron, Norwood, MA, USA) (Figure 2) at a cross-head speed of 1 mm/min. The test was done by using a 4-mm stainless-steel indenter with a rounded tip placed in the central occlusal fossa. All 30 specimens were statically loaded until fracture, and the maximum fracture resistance values were recorded in Newtons by a computer connected to the loading machine.

The data were statistically analyzed using JMP 17 Statistical Discovery from SAS software (SAS, Cary, NC, USA). The Shapiro-Wilk test was used to check the normality of quantitative variables, which were found to be not normally distributed. The Kruskal-Wallis test was used to evaluate differences in fracture resistance between groups, and nonparametric comparisons were made for each pair using the Wilcoxon method at a significance level of α=0.05. Data handling, table creation, and graphics design were performed with Microsoft Office Excel (Microsoft Corporation, Redmond, Washington, United States).

## Results

The fracture resistance (mean ± standard deviation) of the three groups of zirconia surveyed crowns is presented in Table 2. The Kruskal-Wallis test showed statistically significant differences between all the groups (p=0.0183). Zirconia surveyed crowns without a rest seat (control group) presented the highest fracture resistance values (5831± 895.15 N), followed by surveyed crowns with a digitally designed and milled rest seat (5280 ± 1673.33 N). The fracture resistance of these two groups was statistically similar but significantly higher than that of crowns with hand-modified mesial rest seats (4976 ± 322.5 N). A post-hoc comparison using the Wilcoxon method showed that hand-modified rest seat preparation significantly reduced the fracture resistance of the zirconia crowns (Table 3). 

**Table 2:** Fracture resistance of computer-aided design/computer-aided manufacturing zirconia was surveyed on crowns without and with rest seats. *Significant at p<0.05.

Group	Mean (N) ± SD	Confidence Interval		Kruskal-Wallis p-value
		Lower 95%	Upper 95%	
Full contour (control)	5831.46 ± 895.15	4892.0587	6770.868	0.0183*
Milled rest seat	5280.36 ± 1673.33	3524.302	7036.4147	
Hand-modified rest seat	4976.6 ± 322.5	4638.1649	5315.0251	

**Table 3:** Post-hoc comparison using the Wilcoxon method. *Significant at p<0.05.

Level	Level	Score Mean Difference	Standard Error	p-value
Milled rest seat	Hand-modified rest seat	3.83333	2.081666	0.0656
Milled rest seat	Full contour (control)	1.83333	2.081666	0.3785
Hand-modified rest seat	Full contour (control)	-5.83333	2.081666	0.0051*

## Discussion

The purpose of this in vitro study was to determine whether there was a difference in fracture resistance of zirconia surveyed crowns without a rest seat compared to those with a digitally designed, milled, or hand-modified mesial rest seat. Based on the results, our null hypothesis was rejected. Zirconia surveyed crowns with no rest seats, and those with digitally designed and milled rest seats had significantly higher fracture resistance than those prepared with a hand-modified rest seat.

CAD/CAM technology has become very popular in the dental field, with novel digital workflows introduced in pre-doctoral dental education along with traditional technologies [19]. A recent study that surveyed 358 pre-doctoral dental students from different dental schools in North America showed that 87.2% received lectures related to CAD/CAM technology, and 86.9% mentioned having done simulated exercises with CAD/CAM equipment. The authors concluded that students appreciate CAD/CAM technology and believe it makes them better clinicians [20]. Moreover, a recent survey evaluated dentists’ perceptions of chairside CAD/CAM technology using information provided by 114 clinicians, with a general consensus that the overall quality of chairside CAD/CAM restorations is at least as good as those fabricated by a lab technician or much better [21]. The results also demonstrated that CAD/CAM workflows have infiltrated dental practices. 

Currently, tooth-supported all-ceramic restorations can be fabricated from a large variety of materials, such as porcelain, leucite, lithium disilicate, zirconia, and their combinations [22]. Zirconia has been proven to offer high mechanical properties and positive results in clinical studies [23,24]. A recent study evaluated the fracture resistance of monolithic zirconia, veneered zirconia, and metal-ceramic crowns [25]. The highest fracture resistance was observed in monolithic zirconia (4201± 2317 N), followed by metal-ceramic (3609 ± 1210 N) and veneered zirconia (2524 ± 871 N) crown. The study also concluded that monolithic zirconia crowns are highly reliable in terms of load-bearing capacity in the posterior area of the mouth. Another study evaluated the clinical outcomes of monolithic zirconia crowns in posterior teeth with a five-year follow-up by dental students [26]. The study assessed 40 crowns placed on 31 patients, with “excellent” results in 85% of cases, “acceptable” results in 10% of cases, and a “need to be re-done” result in 5% of cases. The study concluded that monolithic zirconia crowns for posterior teeth offer predictable long-term results even when performed by less-experienced clinicians [26]. 

The results of our in vitro study showed that crowns with a rest seat offered lower fracture resistance than crowns without any rest seat, with this finding in agreement with those of other studies. An in vitro study evaluated the fracture resistance of monolithic zirconia crowns without a rest seat and with disto-occlusal, disto-occlusal extended, interproximal, and continuous rest seats for the mandibular right first molar [27]. The significantly lowest fracture resistance was observed for crowns with no rest seat (4238 ± 383 N), followed by continuous rest seat (3601 ± 757 N), disto-occlusal extended (3283 ± 722 N), disto-occlusal (3257 ± 581 N), and interproximal (2723 ± 265 N). Therefore, the authors concluded that the choice of rest seat influences the fracture resistance of CAD/CAM zirconia surveyed crowns. Our study also found that crowns with no rest seat offered the highest fracture resistance (5831 ± 895 N), as compared to crowns with digitally designed rest seats (5280 ± 1673 N) and hand-modified rest seats (4976 ± 322 N) [27].

Unfortunately, no studies have evaluated the effect of hand-modified rest seats for surveyed crowns on fracture resistance. However, some studies have evaluated manual modifications (e.g., creating endodontic access), with results that agree with our findings. A recent study evaluated the effect of manually created endodontic access for anterior crowns on fracture resistance. The study assessed maxillary central incisor lithium disilicate crowns with no endodontic access and with triangular and ovoidal endodontic access; the cemented restorations underwent artificial aging with 10,000 thermal cycles prior to fracturing. The highest values were observed for crowns with no access (1134 ± 127 N), as compared to crowns with triangular (709 ± 75 N) and ovoidal (1000 ± 72 N) endodontic access [28].

The fracture resistance values obtained in this study are greater than the chewing forces reported in the literature. An electromyography and bite-force study of muscular function and dysfunction in masticatory muscles evaluated the maximal effort of patients [29]. Bite force was measured with sensors placed between the first molars, with the results indicating a force of 373 N when chewing almonds and 239 N when chewing gum. Another study also evaluated the maximal bite force for young adults with bruxism, which was measured with a compressive load transducer at the first molar region. A bite force of 806 N was observed among those with bruxism [30].

Care should be taken when clinicians encounter an abutment included in the design of the RPD with an existing zirconia crown. Manually redoing the crown to add a rest seat must be thoughtfully considered. Conservative preparation and prudent planning can save patients costs and time compared to replacing the existing crown with a surveyed crown. However, in cases requiring high levels of preparation leading to cracks or perforation due to limited material thickness, it is wise to use a surveyed crown.

The investigation of only one ceramic material limits this study. Further studies should also evaluate other common ceramics, such as lithium disilicate, and novel hybrid ceramics, such as zirconia-reinforced lithium silicate. Another limitation of the study is that we only evaluated molar crowns; additional information for the posterior region of the mouth can be obtained if premolar crowns are also tested with a similar methodology. Lastly, casting dies were used in this study; it may be interesting to perform a similar study with printed resin dies and natural dentition.

## Conclusions

Based on the results of this study, surveyed crowns without a rest seat displayed higher fracture resistance than those with a rest seat. The fracture resistance of crowns with digitally designed and milled rest seats was statistically similar to that of control crowns without such preparation. Manual rest seat preparation significantly reduced the fracture resistance of the surveyed zirconia crowns.
